# SARS-CoV-2 in Pregnancy: Fitting Into the Existing Viral Repertoire

**DOI:** 10.3389/fgwh.2021.647836

**Published:** 2021-06-24

**Authors:** Roopali Rajput, Jitender Sharma

**Affiliations:** ^1^Independent Researcher, New Delhi, India; ^2^Department of Biochemistry, All India Institute of Medical Sciences, Bathinda, India

**Keywords:** pregnancy, SARS-CoV-2, maternal fetal health, COVID-19, vertical transmission, neonatal infection

## Abstract

The risk of viral infection during pregnancy is well-documented; however, the intervention modalities that in practice enable maternal-fetal protection are restricted by limited understanding. This becomes all the more challenging during pandemics. During many different epidemic and pandemic viral outbreaks, worse outcomes (fetal abnormalities, mortality, preterm labor, etc.) seem to affect pregnant women than what has been evident when compared to non-pregnant women. The condition of pregnancy, which is widely understood as “immunosuppressed,” needs to be re-understood in terms of the way the immune system works during such a state. The immune system gets transformed to accommodate and facilitate fetal growth. The interference of such supportive conversion by viral infection and the risk of co-infection lead to adverse fetal outcomes. Hence, it is crucial to understand the risk and impact of potent viral infections likely to be encountered during pregnancy. In the present article, we review the effects imposed by previously established and recently emerging/re-emerging viral infections on maternal and fetal health. Such understanding is important in devising strategies for better preparedness and knowing the treatment options available to mitigate the relevant adverse outcomes.

## Introduction

Pregnancy leads to numerous physiological changes. Specifically, the immune system undergoes extensive transformation, often termed as becoming “immunocompromised,” but it is nevertheless essential to support the growth of the developing fetus. However, this state is also one where the body is prone to various infections. Moreover, the probable transmission of virus infections from mother to fetus further complicates and aggravates the disease outcome. Such “vertical transmission” may be defined as the spread of any pathogen from a mother to her fetus (antepartum and intrapartum periods) or to a neonate (postpartum period) via the placenta *in utero* through contact with body fluid during delivery or via breastfeeding post-birth (1). It has been evident in several virus infections, such as HIV (2, 3), the Ebola virus (4), the Zika virus (5), etc. Until now, the only antenatal management for viral infections comprises the diagnosis of TORCH (toxoplasmosis, other, rubella, CMV, and HSV type-1 and -2) infections. This panel is now expanded to include: syphilis, listeriosis, parvovirus, coxsackie virus, *Trypanosoma cruzi*, and others (6). However, no specific therapeutic or preventive approach is employed to address the adverse outcomes that may arise from such viral infections. The recent emergence of the potent novel SARS-CoV-2 has further contributed to this fear of vertical transmission and the unknown infection outcomes during pregnancy. The present review is focused on enumerating various viral infections prevalent during pregnancy and the associated risks to maternal and fetal health (Table 1) with special reference to the SARS-CoV-2 infection.

**Table 1:** Various features of virus infections during pregnancy.

**Virus**	**Year of discovery**	**Genome characteristics**	**Prevalence (year of estimation)**	**Signs and symptoms**	**Route of transmission**	**Risk of vertical transmission**	**Mortality rate and impact on infected neonate**	**Mortality rate and impact on maternal health**	**References**
Influenza viruses	Influenza virus first identified in 1933 by Alphonse Raymond Dochez and co-researchers	Segmented -ve sense ssRNA genome; about 13.5 nucleotides	49.1% IAV (2020); 50.9% IBV (2020)	Signs: tachycardia, facial flushing, clear nasal discharge, and cervical adenopathy. Symptoms: Fever, cough, malaise, rhinitis, headache, sore throat, myalgia, nausea, vomiting, otitis, and conjunctiva burning	Respiratory droplets/ aerosols while coughing, sneezing, or any thrustful mouth activities; contact with nasal secretions	High	0.15 deaths per 100,000; Indirect effects to fetus include neurological disorder, leukemia	High mortality rate in pregnancy; Adverse outcomes include pre-term birth, spontaneous abortion; complications increase if co-occurrence of pneumonia: more than 50% of pregnant women with influenza and pneumonia are unable to carry the pregnancy to full term	(7–9)
Cytomegalovirus	Typical signs identified in 1881; CMV isolation and propagation from humans and mice in 1956-1957 by Weller, Smith, and Rowe	Linear dsDNA; 236 kbp	Ubiquitous prevalence- About 50% till the age of 40 years; 100% in Africa and Asia; 80% in Europe and North America	Mild illness with non-specific symptoms, such as fever, sore throat, fatigue, swollen glands; and occasionally, mononucleosis or hepatitis	Contact with infected body fluids, such as saliva, urine, blood, tears, semen, and breast milk	36–65% from 1st to last trimester	Rare; Neurodevelopmental, auditory damage, microcephaly	1 in every 4 pregnant women is infected by CMV; 0.5–2% of all live birth infections	(10–13)
HSV-1	First HSV isolation from fever blister in 1919 by Lowenstein	Linear; dsDNA; 152 kbp	12.1% (2016)	Sores around the mouth and lips	Contact with infected lesions, mucosal surface, or via genital or oral secretions	Medium during late pregnancy; Low during early pregnancy	80%; Localized skin, eye, and mouth (SEM), central nervous system (CNS) with or without SEM or disseminated disease; major impact: blindness, seizures, and learning disabilities	Spontaneous abortion, intrauterine growth restriction, preterm labor, and congenital and neonatal herpes infections	(14–18)
HSV-2		Linear; dsDNA; linear; 154.7 kbp	48.1% (2016)	Sores around genitals or rectum					
Varicella zoster virus	First isolated in 1954 by Thomas Huckle Weller	Linear dsDNA; 125 kb	97% decline in VSV infections since pre-vaccine era from 1993–1995 to 2013–2014 in the U.S.	In children, rash on scalp, face, and trunk are the first signs, followed by rash on extremities, fever, malaise, headache. In adults, fever and malaise for initial 2 days of infection followed by appearance of rash	Contact with infected lesion fluid; person-to-person	High in 8–20 weeks of gestation	30%; congenital varicella syndrome	10–20% VSV infections during pregnancy are accompanied by pneumonia, which may cause up to 40% mortality	(19–21)
Hepatitis C virus	1987 by Michael Houghton, Qui-Lim Choo, George Kuo, and Daniel W. Bradley; 1988 by Harvey J. Alter and his team	ssRNA; positive-sense; 9600 nucleotides long	1% viraemic prevalence accounting for 71.1 million cases (2015) with genotypes 1 and 3 being the most common; 2.8% (sero-prevalence as per systematic review, 2013)	Acute hepatitis C usually shows no signs/symptoms, but may exhibit: jaundice, along with fatigue, nausea, fever, and muscle ache; Chronic hepatitis C is generally a silent infection not causing any disease until the liver gets substantially damaged and leads to easy bleeding and bruising, fatigue, poor appetite, jaundice, dark-color urine, itchy skin, ascites, swelling in legs, weight loss, confusion, drowsiness and slurred speech (hepatic encephalopathy), spider-like appearance of blood vessels on skin (spider angiomas)	Infected blood	5.8%; With higher risk in case of co-infection with HIV (10.8%)	Preterm birth, late neonatal death	Intrahepatic cholestasis pregnancy	[(22, 23), https://www.mayoclinic.org/diseases-conditions/hepatitis-c/symptoms-causes/syc-20354278]
Hepatitis E virus	1978	ssRNA, positive-sense; 7.2 kb	About 20 million cases including 3.3 million symptomatic infections per year; 3.3% mortality estimate (2015)	Acute hepatitis E shows no signs/symptoms; Chronic disease exhibits: fever, fatigue, loss of appetite, nausea, vomiting, abdominal pain, jaundice, dark color urine, clay-color stool, pain in the joints	Fecal-oral route	High during the second and third trimester	High perinatal morbidity and mortality	20–25% mortality in pregnancy during the third trimester; Fulminant hepatitis, acute liver failure, death	[(24), https://www.cdc.gov/hepatitis/hev/hevfaq.htm; https://www.who.int/news-room/fact-sheets/detail/hepatitis-e#:~:text=Hepatitis%20E%20is%20a%20liver,of%20hepatitis%20E%20(1)]
HIV	First isolated and identified by Luc Montagnier's team (Luc Montagnier and Françoise Barré-Sinoussi received the Nobel Prize in 2008)	two copies of +ve sense ssRNA; 9,200–9,800 nucleotides	0.8% in adults (2018); 75.7 million diagnosed HIV+ since 1981; 21% unaware of their HIV status	Non-specific symptoms, such as fever, lymph node enlargement, fatigue, malaise, rash with small, only slightly raised lesions, and/or gastrointestinal symptoms	Blood or transplanted organs, including bone, vertical transmission, breast milk	>90% in late pregnancy	0.04–0.094%; If left untreated- Repeated fungal mouth infections (thrush); Poor weight gain; Enlarged lymph nodes; Neurological problems; Multiple bacterial infections (i.e., pneumonia)		(25–29)
Lassa virus	1969	Two ssRNA segments; 10.4 kb combined length (short strand: 3.4 kb; long strand 7 kb)	0.1 million- 0.3 million cases per year including about 5,000 deaths per year in the west of Africa	Mild in 80% cases: fever, malaise, weakness, and headache; Serious in rest of the 20% cases: hemorrhage in gums, eyes, or nose, etc., respiratory distress, frequent vomiting, facial swelling, chest- back- and abdomen- pain, shock, neurological symptoms, like, loss of hearing, tremors, and encephalitis. Multi-organ failure in more severe cases leading to death	Zoonotic transmission: via excretions of infected rodent multi-mammate rat, *Mastomys natalensis*; Human-human transmission via contact with body fluids of the infected person	High risk due to high viral load in the placenta and maternal blood	Premature birth Note:-More evidence with well-planned studies is required, although the risk is high due to serious outcomes of infection and high viral load in infected maternal and fetal tissues	high viral load in the placenta, fetal tissue, and maternal blood impose adverse outcomes	https://www.cdc.gov/vhf/lassa/pdf/factsheet.pdf
Zika virus	1947	Positive sense; ssRNA; about 11 kb	27% confirmed cases out of 0.7 million suspected cases in America (2015–2017)	Mostly asymptomatic; otherwise mild clinical features that are typical of maculopapular rash, like, fever, arthralgia, non-purulent conjunctivitis	Through infected *Ae. aegypti* and *Ae. Albopictus* vectors	47, 28, and 25% in first, second, and third trimesters, respectively	Congenital microcephaly, serious brain anomalies, Guillain-Barré syndrome, rare cases of encephalopathy, meningoencephalitis, myelitis, uveitis, paresthesia, and severe thrombocytopenia	Fetal loss, IUGR pregnancies	(30–32)
Ebola virus	1976	Non-segmented; negative sense RNA; 18–19 kb	28,000 confirmed cases and 11,000 deaths (2013–2016); case fatality rate 25–90% (average nearly 50%) as per previous outbreaks	Sudden symptoms: include fever, fatigue, muscle pain, headache, and sore throat; Other symptoms: vomiting, diarrhea, rash, impaired kidney and liver function, internal and external bleeding, like, gum-bleeding or blood excretion in stool	Zoonotic transmission: via contact of blood, body fluids/secretions, tissues, etc. of infected fruit bats, chimpanzees, gorillas, monkeys, forest antelope, or porcupines; Human-human transmission: direct contact via broken skin tissue or mucous membranes	Pregnant women recovered from acute Ebola may express virus in breastmilk, or in pregnancy-related body-fluids and tissues, which exhibits a high risk of transmission to baby and to others.	Preterm labor and spontaneous abortion	Vaginal and uterine bleeding causing maternal death	[(33), https://www.who.int/news-room/fact-sheets/detail/ebola-virus-disease]
SARS-CoV-2	Identified in 2020 by the China Novel Coronavirus Investigating and Research Team and named by the Coronaviridae Study Group of the International Committee on Taxonomy of Viruses in the same year	+ve sense ssRNA; 29,811 nucleotides	79.2 million cases and over 1.7 million deaths since onset of the pandemic in 2019	Major: fever, dry cough, tiredness; Minor: aches and pains, sore throat, diarrhea, conjunctivitis, headache, loss of taste or smell, a rash on the skin, or discoloration of fingers or toes Occasional serious symptoms: difficulty breathing or shortness of breath, chest pain or pressure, loss of speech or movement	Respiratory droplets/aerosols; fomites	2–3.9%; however true estimate yet known	True estimate not known yet; preterm labor and delivery, premature rupture of membranes, low birth weight, intrauterine fetal distress and growth restraint, feeding intolerance, asphyxia, pneumonia, and respiratory distress	Preterm labor and delivery, premature rupture of membranes	(1, 34, 35)

## Long-Established Viral Infections During Pregnancy

Over the years, the risk of viral pandemics has grown with evolving human activities. The first two trimesters of pregnancy exhibit increased inflammatory responses, but the third trimester is a phase of lower immunological activity (36, 37). Encountering any infectious pathogen makes things difficult for both the mother-to-be as well as the to-be-born baby. Progressing time demands a precise understanding of the viral diseases that may hold the potential for major outbreaks. The past pandemics caused by the influenza, Ebola, and Lassa viruses (38, 39) have shown pregnant women to be vulnerable targets with high incidences of fatality and disease severity (40, 41).

### Influenza Viruses

Of the four (A, B, C, and D) types of influenza viruses, types A and B are known to cause mild to severe disease in humans. While, both A and B influenza viruses cause seasonal epidemics, pandemics are caused by only the influenza A viruses (IAVs) that are further classified into subtypes on the basis of two viral surface proteins, i.e., hemagglutinin (H) and neuraminidase (N) (42). At present, 18 different H subtypes (designated as H1-H18) and 11 different N subtypes (designated as N1-N11) have been identified. Given these many subtypes of H and N, there can be a total of 198 combinations for possible influenza A virus variants, however, till now only 131 IAV subtypes have been identified (42). IAV (H1N1) and IAV (H3N2) are the most commonly circulating seasonal subtypes. Since the 2009 swine flu pandemic, the pH1N1 strain has undergone mutations and adapted itself as a seasonal IAV(H1N1) strain (42).

#### Risk to the Mother

Nevertheless, for pregnant women, the risk of severe illness or death from seasonal or pandemic strains of influenza viruses remains high (43). As evident during the 1918 and 1957 flu pandemics, pregnant women exhibited high mortality rates (44, 45) and adverse pregnancy outcomes like spontaneous abortion and preterm birth (44, 46). More than 50% of influenza-infected pregnant women with pneumonia could not successfully carry the pregnancy to term (44) during the 1918 pandemic. On the other hand, the 1957 Asian flu witnessed birth defects, spontaneous abortions (47), fetal death, and preterm delivery (48). Seasonal influenza virus infections, monitored over 19 influenza seasons, exhibited a significantly higher likelihood of hospitalization of pregnant women with cardiopulmonary indications (49). Acute respiratory disease, asthma, or other underlying conditions further make pregnant women vulnerable to influenza viruses during the flu season (50, 51), and they are thus recommended for influenza vaccination.

#### Risk to the Developing Fetus

Although viremia is quite uncommon in influenza, it has been associated with severity of disease following infection with pH1N1/09 strain (52). Vertical transmission also seems to be a rare event (53); however, mouse model studies suggest possible adverse effects, as evident by histopathological alterations in the brain (54) or behavioral changes (55) in the progeny. Maternal influenza infection has also been linked to childhood leukemia (56), schizophrenia (57), and Parkinson disease (58). Overall, influenza viruses seem to project indirect effects on the developing fetus.

### Cytomegalovirus

Cytomegalovirus is the major cause of infection during pregnancy, being responsible for infecting one in four pregnant women and 0.5–2% of all live birth infections (13). The incidence rate of CMV infection in women of a reproductive age belonging to developed and developing nations is 60 and 90%, respectively (59). The development of anti-CMV antibodies in the mother is crucial in overcoming CMV infection; however, reactivation of the virus may occur in 10% of seropositive women. The highest occurrence rate of CMV may be attributed to the multiple pathways employed by the virus to gain entry into the host (60). The various cell types prone to CMV infection include epithelial cells, endothelial cells, muscle cells, fibroblasts, trophoblasts, and monocytes/macrophages, human neuronal cells (61). Although the disease severity is unaffected during pregnancy, CMV is known to impose serious implications during such states.

#### Risk to the Mother

Antepartum maternal infection remains mostly undetected due to non-specific symptoms and only mild febrile illness. Once the mother is infected, CMV may be transmitted to the fetus either through the placenta or via ingestion or aspiration of cervicovaginal secretions during delivery, breastfeeding, or rarely while ascending from the genital tract of the infected mother.

#### Risk to the Developing Fetus

The most common adverse fetal outcomes include congenital viral infection, which occurs in about 0.5% of cases (62). The primary viral targets include the ventricle, Organ of Corti, and neurons of the eighth cranial nerve, leading to congenital hearing loss (63). The rate of vertical transmission increases with progressing gestation with 36.5% during the first trimester, 40.1% during the second trimester, and 65% during the third trimester (17, 64), while, interestingly, the disease severity decreases with the increasing gestational age (65, 66). The infected neonates remain largely asymptomatic and start exhibiting neurodevelopmental damage within the first 3 years of age (67).

### Herpes Simplex Virus

HSV-1 and HSV-2 have a combined seroprevalence of 72% in pregnant women (68). The most common STD is the genital herpes simplex virus (HSV-2) infection in adult females with an estimated 16% incidence in male and female combined and detection of almost 0.8 million new cases every year (21).

#### Risk to the Mother

As per the National Health and Nutrition Examination Surveys (NHANES), the incidence of HSV-2 infection is greater in women (23.1%) than men (11.2%) (40). Ethnicity, financial well-being, cocaine abuse, onset of sexual activity, sexual behavior and number of partners, and the presence of bacterial vaginosis all seem to affect women's vulnerability to HSV before pregnancy (69). HSV infection during pregnancy causes spontaneous abortion, intrauterine growth restriction, preterm labor, and congenital and neonatal herpes infections (70).

#### Risk to the Developing Fetus

The development of anti-HSV antibodies seems to have no effect on neonates. However, the risk of neonatal herpes infection increases with the gestational period from <1% during early pregnancy to up to 50% during late pregnancy (21, 71). The reason for this may be the inability of antibody development before labor to enable inhibition of HSV replication and shedding. Transplacental vertical transmission is rare, and 80–90% of perinatal transmission happens during delivery (17). HSV infection in neonates may affect localized skin, eye, and mouth (SEM) and the central nervous system (CNS) with or without SEM or disseminated disease. The untreated latter cases exhibit high (80%) mortality (14–17). The major neurological defects in infected neonates include blindness, seizures, and learning disabilities.

### Varicella Zoster Virus

VSV infection causes chickenpox, which is a common but highly contagious illness primarily experienced during childhood. The major delinquent manifestation is the development of maculopapular to vesicular rashes all over the body. The virus gets transmitted through aerosols and close contact (72). An initial varicella zoster infection following chickenpox may lead to the latent stay of the virus in dorsal root ganglia for years and may reactivate as herpes zoster during adulthood. Although VSV infection occurs during childhood and most women of reproductive age have developed considerable immunity against it, it may still occur in 0.7/1,000 pregnancies.

#### Risk to the Mother and the Developing Fetus

Primary VSV infection in pregnant women causes considerable maternal and fetal morbidity and mortality. Although the pediatric infection is self-limiting, 10–20% of VSV infections in pregnancy are accompanied by pneumonia, a condition that may lead to up to 40% fatality (21). Fetal morbidity and mortality are linked to the development of congenital varicella syndrome, which may occur in 0.4–2% VSV-confirmed pregnancies within the initial 20 weeks of gestation (73). The syndrome is characterized by limb hypoplasia, microcephaly, hydrocephaly, cataracts, intrauterine growth restriction, and mental retardation (74) and is believed to be caused due to *in utero* VSV reactivation instead of primary fetal infection (75).

### Hepatitis C Virus

#### Risk to the Mother

HCV poses a severe threat to pregnant women with adverse fetal outcomes, such as preterm birth (76), late neonatal death (77), and intrahepatic cholestasis of pregnancy (the risk of which increases if the mother has been HCV positive before pregnancy) (78). Much of the disease burden data come from the United States, where the elevated incidence of HCV aligned with the opioid epidemic. The HCV burden increased in IDU-associated HCV cases in the younger population, consisting of women who were pregnant and of reproductive age (79). There are geographical inequities in HCV healthcare management, and the prevalence thus varies across the globe. The incidence of HCV cases elevated from 1.8 to 4.7 per 1,000 live births according to a recent analysis of the National Center for Health Statistics data (80). As per the CDC, the rate of HCV infection increased by over 400% (0.8 to 4.1 per 1,000) in cases of women where live births were recorded. In pregnant women belonging to the European Union/European Economic Area, the HCV incidence is 0.1–0.9% (81) while that of Africa is 3.4% (82).

#### Risk to the Developing Fetus

Vertical transmission of HCV is estimated at 5.8% with a significantly higher rate among HIV-co-infected (10.8%) pregnancies (83). In the US, the incidence is 3.6% (84), whereas in Spain it is 7% (85) in HCV/HIV co-infected pregnant women. In spite of these statistics, the true estimate is still a challenge owing to the availability of scarce information on the subject and also the substantial time gap between childbirth and testing for HCV antibodies (currently recommended at ≥18 months of age), which may lead to loss of contact with the cases to be examined (79). The mode of delivery or breastfeeding does not appear to impact the risk of HCV transmission, while the prolonged duration of ruptured membranes may be a risk factor for the same (86). The association between the viral load and vertical transmission of HCV is under speculation, but conclusive recommendations cannot be made due to insufficient data (79).

### Hepatitis E Virus

Hepatitis E Virus is the major cause of self-limiting acute viral hepatitis in healthy adults and chronic viral hepatitis in immunocompromised individuals.

#### Risk to the Mother

There have been several investigations to understand the effects of HEV infection on maternal and fetal health; however, definitive conclusions cannot be made due to contradictory observations among the different studies (87–90). In a recent 5-years single-center study in India, 1,088 patients (550 pregnant and 538 non-pregnant controls) were evaluated to understand the course and severity of HEV infection during pregnancy (91). All the patients were confirmed for either acute viral hepatitis (AVH) or acute liver failure (ALF) through clinical examination and biochemical investigations. The HEV infection was observed in 80.36% of pregnant women, with 73.38% prevalence in ALF cases alone. Also, the mortality rate was recorded at almost 76% due to HEV-infection in the studied subjects (91). Other studies suggest fulminant hepatitis failure with a mortality rate of up to 30% in pregnant women due to HEV infection (88, 90, 92).

#### Risk to the Developing Fetus

Vertical transmission of HEV with significant perinatal morbidity and mortality has been reported (90, 93, 94), and ribavirin and IFN-α administration is thus not carried out during pregnancy due to the risk of birth defects (42, 95, 96). The adverse fetal outcomes include preterm labor (97, 98) and disseminated intravascular coagulation (DIC) (93).

### Human Immunodeficiency Virus/Acquired Immunodeficiency Syndrome

HIV/AIDS continues to be the worst pandemic ever with 75.7 million individuals diagnosed with HIV infection and about 32.7 million deaths between 1981 and up until the end of 2019 (99). Globally, in 2019, women represented almost 48% of the total new HIV cases, and, likewise, in 2018, the incidence of new HIV cases in women aged 15–24 years was 55% higher as compared to men of the same age range. While the majority of adult women contract HIV infection via heterosexual contact, most childhood infections are caused due to vertical transmission (100). Almost 90% of untreated HIV cases proceed to develop AIDS and subsequent death with opportunistic infections due to the significant reduction in CD4 T cells. However, with significant improvements in medical care management of HIV cases, the life expectancy can be increased by as long as 15 years through the administration of anti-retroviral therapy.

#### Risk to the Mother and the Developing Fetus

Vertical transmission accounted for about 180,000 global new HIV infections in 2017 (100). Fortunately, with advanced healthcare systems and the use of cART, rates of perinatal transmission have reduced, leading to a smaller number of childhood exposures progressing into full-blown AIDS. Although the development of illness following HIV infection is not affected by the pregnant state, the risk of mother-to-child transmission forms a major concern. Vertical transmission of HIV can occur during intrauterine life, delivery, or breastfeeding. The adverse fetal outcomes include preterm birth, low birth weight, small size for gestational age, and stillbirth (101).

### Lassa Virus

The Lassa virus causes Lassa fever, or Lassa hemorrhagic fever (LHF), an acute viral hemorrhagic fever that was first identified in 1969 in Lassa, Nigeria (96) and is transmitted through “multimammate rat” (*Mastomys natalensis*).

#### Risk to the Mother

The illness is endemic and a major cause of mortality for pregnant women in the West African regions, viz., Sierra Leone, Liberia, Guinea, and Nigeria (102). Fascinatingly, in cases of Lassa virus infection, maternal health rapidly improves as soon as the fetus is removed from the uterus either by spontaneous abortion or delivery (38). Also, the mortality is higher in cases of non-evacuated uterus (10/26 fatal outcomes) as compared to the cases where delivery was ensured (4/39 fatalities) (38). This may be due to placenta-mediated regulation of the maternal immune system. Even if the placenta is not directly infected, it is capable of responding to invading pathogens and hence seems to be a crucial regulator of a pregnant woman's response to virus infection (103). High viral load in maternal blood, placenta, and fetal tissue accounts for higher mortality rates of pregnant women than the non-pregnant counterparts (104). Maternal mortality risk increases with the progressing gestational period: from 7% during the first two trimesters to as high as 30% in the last trimester. Almost 50% mortality has been recorded within a month post-partum in contrast to 13% in non-pregnant females (104).

### Risk to the Developing Fetus

Lassa virus infection is speculated to impose adverse outcomes to the developing fetus, given the high viral load in placenta, fetal tissue, and maternal blood. However, due to extremely limited evidence on clinical characteristics and course of Lassa fever in pregnancy and variability in study design and methodologies employed, the exact maternal and perinatal outcomes cannot be convincingly described (105). The vertical transmission and premature birth have been reported, though.

## Re-Emerging and Less Understood Viral Threats

### Zika Virus

The unexpected re-emergence of the Zika virus in 2015 and the subsequent outbreak in Brazil reinforced that the management of viral infections is of utmost significance during pregnancy.

#### Risk to the Mother and the Developing Fetus

Zika virus is transmitted by mosquitoes and potentially via a sexual route (106).

What was previously known to progress from fever and rash to Guillain-Barre syndrome is now reported to cause fetal brain and CNS anomalies in neonates born to Zika-virus-infected women (95, 107), as confirmed by the presence of viral nucleic acid in amniotic fluid (108). The adverse fetal outcomes include microcephaly, abortions and IUGR pregnancies, and other complications. Viral load in amniotic epithelia during mid-gestation is reported to be higher than in the late-gestation period (109). Cytotrophoblasts were also observed as viral targets, and, during early gestation, they were linked to loss of proliferation. Such a condition may explain the miscarriage and growth restriction outcomes.

### Ebola Virus

Ebola virus, the cause of Ebola Hemorrhagic fever (EHF), primarily causes human infections in Africa (110). However, with extensive global travel, infectious nature of the pathogen, and potential effects on maternal and fetal health the virus has become a global threat. Although quite uncommon, the Ebola virus has imposed severe illness and considerable EHF outbreaks in Africa. The causative species, *Zaire ebolavirus*, was identified in 1976 in Kikwit and, during the then epidemic incidence of Ebola virus infection, saw a higher incidence among women than men (111, 112). Also, mortality was higher in pregnant than non-pregnant women. The 1976 Ebola epidemic witnessed 46% infections and 89% mortality among pregnant women.

#### Risk to the Mother

One of the main manifestations during the 1976 epidemic was vaginal and uterine bleeding leading to the death of infected pregnant females (93% mortality rate) within 10 days of onset of symptoms (113, 114).

#### Risk to the Developing Fetus

The adverse fetal outcomes of EHF during pregnancy include preterm birth and abortion. The 1976 epidemic led to a 23% incidence of spontaneous abortions of pregnancies, while the same was considerably higher (67%) in the 1995 epidemic (113, 114).

### SARS-CoV-2

Adverse outcomes of pregnancy have been reported following infection with the previous SARS- and MERS- coronaviruses (15, 51, 86); hence the current pandemic SARS-CoV-2 aggravates the apprehension related to maternal and fetal well-being. Initially, there have been fears associated with SARS-CoV-2 led pregnancy complications and adverse fetal outcomes (26, 28, 115, 116). Further studies with large sample sizes (116 in China and 427 in the UK) ruled out such apprehensions, however, and demonstrated a higher rate of cesarian section deliveries (4, 104, 117, 118).

#### Risk to the Mother

Few studies suggest mild illness in COVID-19 confirmed pregnant females with lower mortality than the non-pregnant COVID-19 patients (119) and premature delivery as a major adverse fetal outcome (119). As per a month's rigorous surveillance in Sweden, a requirement of intensive care for COVID-19 confirmed pregnant and early postpartum women has been reported (120) at a relative risk of 5.4 (95% CI, 2.89–10.08). The patients also required invasive mechanical ventilation with a relative risk of 4.0 (95% CI 1.75–9.14) in contrast to the non-pregnant women of similar age. An expansion of the denominator to include 50% more pregnancies (possible miscarriages and early intrauterine fatalities), still exhibited a high RR 3.5 (95% CI, 1.86–6.52). These findings, although are from only 53 patients aged between 20 and 45 years without any information on co-morbidities, reflect the need to focus and further study the possible risks associated with SARS-CoV-2 infection in pregnant women (120). Another recent study aimed at investigating the outcomes of pregnancy and analysis of the clinical features in COVID-19 confirmed pregnancies vs. non-pregnant cases highlighted the worsening of morbidity with the progression of pregnancy due to SARS-CoV-2 infection. The mortality rate seemed to be unaffected in this retrospective analysis. The study analyzed the record of 188 pregnant cases and 799 age-matched non-pregnant counterparts from four tertiary care hospitals in Turkey. The severity of SARS-CoV-2 infection was significantly high in pregnant women especially at >20 weeks of gestation (*p* < 0.001). In comparison to non-pregnant cases, pregnant cases displayed significantly high frequency of oxygen support (10.1 vs. 4.8%; *p* ≤ 0.001), intensive care unit admission (3.2 vs. 0.6%; *p* = 0.009), presence of fever (12.8 vs. 4.4%; *p* < 0.001), tachypnea (7.0 vs. 2.4%; *p* = 0.003), and tachycardia (16.0 vs. 1.9%; *p* < 0.001). Co-morbidities were present in 14.4% of pregnant women. Of the 188 pregnant cases, about 32% delivered (18.3% vaginal and 81.7% cesarean) during the SARS-CoV-2 infected state, with 66.7% at <37 weeks of the gestation period (121). Similar to Turkey, maternal mortalities have not been associated with SARS-CoV-2 infection in China (117, 122, 123). However, deaths have been reported from developing as well as developed nations (124, 125).

Although, under-reporting of maternal deaths due to COVID-19-related complications is highly likely, another issue while elucidating SARS-CoV-2 infected pregnancies is the lack of true denominator value. A possible solution may be to include the entire pregnancies in investigations, but the use of sophisticated and expensive techniques, like real-time RT-PCR for confirmatory diagnosis of SARS-CoV-2 infection, poses limitations. Reliable serological assays become when available may aid in resolving this constraint. Assessment of seroconversion rates in stratified unselected samples can be another approach (given the fact that blood specimens are commonly collected from pregnant women for routine antenatal investigations). Nevertheless, at present, robust estimates of COVID-19 severity and risk of morbidity and mortality in pregnant women are needed. For this, large-scale analysis from different geographical regions is required. Converging data from different countries would be necessary to neutralize the effects of confounding factors and outcome modifiers. Simulation/ prediction models are only assumptions and hence, accurate clinical data with rigorous collection protocols, although more nuanced, would enable the generation of real scenarios. Although analysis and conclusions made out of small-scale uncontrolled studies need to be done cautiously, the risk of COVID-19 in pregnancy cannot be avoided.

#### Risk to the Developing Fetus

One of the major concerns of the medical fraternity during COVID-19 has been the vertical transmission of the SARS-CoV-2 infection and the adverse fetal outcomes. Although, there have been no reported cases of vertical transmission from SARS-/ MERS-CoV infections, the fear existed in the case of SARS-CoV-2. The SARS-CoV-2 gains entry into the host cell by binding to the angiotensin-converting enzyme 2 (ACE2) receptor, which is present in the placenta (126) and also expressed in syncytiotrophoblast, cytotrophoblast, endothelium, and vascular smooth muscle cells from both primary and secondary villi (127). Furthermore, there have been reports suggesting the presence of ACE2 in female reproductive organs, viz., ovary, uterus, and vagina (128). In a nutshell, the ACE2 receptor is expressed in a variety of tissues involved throughout a pregnancy period. A recent single-cell RNA sequencing investigation demonstrated ACE2 expression in the cells (stromal, perivascular, placental, and decidual) at the maternal-fetal interface (129). Another similar investigation by single-cell RNA sequencing highlighted the limited co-expression of ACE2 with the TMPRSS2 in placental cells throughout the pregnancy period, however, suggested viral entry into placenta cells via ACE2 and a non-canonical cell-entry mediator (130).

The adverse fetal outcomes include preterm labor and delivery, premature rupture of membranes, low birth weight, intrauterine fetal distress and growth restraint, feeding intolerance, asphyxia, pneumonia, and respiratory distress. Such observations require further confirmatory studies, though. In one such case, a COVID-19 confirmed mother with severe complications, i.e., pneumonia with mechanical ventilation support, ECMO (extracorporeal membrane oxygenation), and MODS delivered a stillborne infant who was negative for SARS-CoV-2 infection (37).

With regard to vertical transmission, different studies involving patients in China, indicated an overall 2% (8/397) incidence of mother to fetus transmission of the virus (1). Yan and colleagues investigated 116 SARS-CoV-2 infected pregnant women (forming one of the largest cohorts) in China. Of the 100 neonates, 86 were sampled for nasopharyngeal swab (NPS) testing, and none of them were positive for the SARS-CoV-2 virus (117). Another large cohort study conducted in the United Kingdom investigated 427 pregnant COVID-19 patients. Of the 244 neonates sampled for NPS, 12 were positive for the SARS-CoV-2, indicating a 4.9% rate of vertical transmission (118). In another analysis done in a hospital located in New York, it was found that none of the 48 newborns, who were tested on the same day of birth, were positive for SARS-CoV-2 (131). In Italy, 3 out of 42 newborns, within 48 h of birth, exhibited positive SARS-CoV-2 NPS tests (132). As per the current data, a 3.5% (19/539) incidence of SARS-CoV-2 vertical transmission can be recorded (1) for the neonates tested outside China. As per a meta-analysis, of 38 cohort/case studies, performed by Kotlyar and the research group, an overall 3.2% pooled proportion tested positive for viral RNA in NPS of newborns sampled right after or within 48 h of birth (1). In another analysis, a rate of 3.91% was indicated for vertical transmission of the SARS-CoV-2 based on viral RNA positivity (119). In an interesting study on a neonate born to a COVID-19 confirmed mother, anti-SARS-CoV-2 IgG and IgM antibodies were detected 2 h after delivery. Also, the infant showed elevated levels of cytokines, however, the viral RNA could not be detected in NPS, placenta, umbilical cord blood, amniotic fluid, maternal blood, vaginal secretions, or even breastmilk (133). Few initial investigations done in China demonstrated the presence of anti-SARS-CoV-2 IgM antibodies in neonates born to COVID-19 confirmed mothers (134, 135), suggesting *in utero* viral transfer of IgM cannot pass through the placenta.

## Changes in Maternal Immune System During Pregnancy

The onset of pregnancy imposes considerable challenges to maternal health. Right from the beginning, the pregnant female encounters risk from as close as the paternal alloantigens (expressed by both fetus and placenta). Fortunately, mother nature has taken care of the mothers-to-be, and, thus, a classic response to the paternal alloantigens is not observed in general (136); the alloantigens may retain maternal blood and tissue for a prolonged duration even after childbirth (137).

During pregnancy, circulation of monocytes, granulocytes, pDCs, and mDCs increases in blood with peaks occurring in two trimesters, with a parallel decrease in CD3, CD4, and CD8 T cells, as compared to the post-partum period. While the number of B cells declines in the third trimester, NK cells CD56 decline in the last two trimesters of pregnancy. The latter is also the time when levels of IFN-γ, TNF, and IL-6 decline in contrast to the post-partum period (138); however, these observations are contradictory (139). The number of maternal monocytes remains unaffected; instead, phenotypic alterations have been noted, such as elevated expression of CD11a, CD11b, CD54, and CD64 (139). By the 13th week of gestation, the maternal PBMCs begin to harbor phenotypic and functional modifications. Elevated secretion of IL-1β and IL-12 with a simultaneous decline in TNF-α is observed (140).

### Maternal-Fetal Interface

The maternal-fetal interface is essentially formed by the placenta, which develops from the uterine wall and is capable to express various receptors and micro-vesicles (141). The placenta connects and provides hormonal, nutritional, and oxygen supply to the developing fetus while also moderating the mother's immune responses (142). Decidual cells, uterine NK cells, DCs, and Tregs form at the maternal side of the placenta, while the placental villus (fetal blood vessels along with fibroblasts) and placental villous macrophages are of fetal origin, and Hofbauer cells constitute the fetal side of the placenta (61, 143). The number of regulatory T cells (Tregs) increases in pregnancy, specifically in peripheral, deciduous, and umbilical cord blood (144). Such elevated numbers are important as the Tregs stimulate expression of IL-10 and TGF-β, which, in turn, modulates CD4+ T and CD8+ T lymphocyte levels during pregnancy (145).

Furthermore, in the early pregnancy period, NK cells (contributing to about 70% of decicuous leukocytes) are accumulated at the maternal-fetal interface in early pregnancy (146). Such an interesting feature is important since the NK cells modulate the release of cytokines and chemokines, control the invasion of trophoblasts, and warrant a sufficient supply of maternal blood (143, 146, 147). Also, changes in the hormone levels regulate maternal immune responses during pregnancy, for instance, the number of DCs and monocytes reduces, activation of macrophages, T, and B cells also declines (148). Estrogen reportedly stimulates Foxp3 Tregs in order to efficiently establish a tolerogenic milieu (149).

## Immunological Account of SARS-COV-2 Infection During Pregnancy

Serological investigations have indicated incidence of lymphopenia, neutrophilia, elevated CRP (C-reactive protein) (133, 150), ALT, AST, and D-dimer upon SARS-CoV-2 infection during pregnancy (133, 135, 151). In a study, few COVID-19 positive cases developed anemia and dyspnea (152). Another report highlighted the potential effects of altered calcium and albumin levels in the severity of SARS-CoV-2 infection during pregnancy (153, 154). Low platelet count has been linked to COVID-19 related deaths in pregnancy (155, 156). In spite of the indications from few cases, conclusive statements cannot be made on the effects exerted by SARS-CoV-2 on maternal and fetal health due to limited evidence. Long-term effects of stress and physiological temperature control in COVID-19 confirmed pregnancies are speculated. Elevated IL-10 levels in COVID-19 confirmed mothers may modulate inflammation and sustain pregnancy (64, 157).

A noteworthy observation following the 2009 influenza pandemic was a decline in cytokine response to bacterial infections. This is finding is crucial as it indicates that SARS-CoV-2 infection may also cause impaired immune responses to any other future infections or even insufficient immunological responses to vaccines.

## Conclusion

Although, the role of virus infections in increasing morbidity and mortality during pregnancy is well-perceived, yet limited information is available on the mechanism of pregnancy-led maternal responses to viral invasion. The emergence of the current pandemic virus strain, i.e., SARS-CoV-2, has spanned a year; little is known about its pathogenesis, clinical signs, and symptoms, disease course, and the adverse outcomes it may have on maternal/fetal health. Furthermore, harboring of different mutations as per the geographical regions (158) suggests the adaptation of the SARS-CoV-2 and its continued circulation among humans. Dedicated studies involving appropriate methodology, controls, effective sample size, and assessment of various parameters need to be performed to ascertain the exact effects of the SARS-CoV-2 infection during different trimesters of pregnancy, effects of cytokine storm to neonatal well-being, and other potential implications. This would be essential in defining guidelines for mandatory testing and post-diagnosis perinatal care.

## Author Contributions

RR conceived, conceptualized, searched, analyzed the information/data, drafted the initial manuscript, and reviewed the final version. JS provided considerable intellectual input, analyzed the data, and reviewed the final version of the manuscript. All authors contributed to the article and approved the submitted version.

## Conflict of Interest

The authors declare that the research was conducted in the absence of any commercial or financial relationships that could be construed as a potential conflict of interest.
